# Passive Measures of Physical Activity and Cadence as Early Indicators of Cognitive Impairment: Observational Study

**DOI:** 10.2196/72946

**Published:** 2025-09-12

**Authors:** Huitong Ding, Stefaniya Brown, David R Paquette, Taylor A Orwig, Nicole L Spartano, Honghuang Lin

**Affiliations:** 1 Department of Anatomy and Neurobiology, Boston University Chobanian & Avedisian School of Medicine, Boston, MA, United States; 2 The Framingham Heart Study, Boston University Chobanian & Avedisian School of Medicine, Boston, MA, United States; 3 Section of Endocrinology, Diabetes, Nutrition, and Weight Management, Boston University Chobanian & Avedisian School of Medicine, Boston, MA, United States; 4 Massachusetts AI and Technology Center, Manning College of Information and Computer Sciences, University of Massachusetts Amherst, Amherst, MA, United States; 5 Department of Medicine, University of Massachusetts Chan Medical School, S6-755, 55 Lake Avenue North, Worcester, 01655, United States, 1 7744554881

**Keywords:** passive physical activity, wearable device, older adults, cognitive impairment, neuropsychological test, physical activity

## Abstract

**Background:**

Emerging research suggests that regular physical activity can reduce the risk of cognitive decline. However, most prior studies rely on self-reported measures, which are subject to recall bias, subjectivity, and limited temporal resolution.

**Objective:**

This study aims to investigate whether objectively measured physical activity, captured through passive accelerometry, is associated with incident cognitive impairment and neuropsychological test performance and to evaluate its potential as an early indicator of cognitive decline.

**Methods:**

We analyzed data from the Framingham Heart Study (FHS), a community-based cohort with longitudinal surveillance of cognitive impairment. Participants wore an Actical accelerometer for at least 3 days (excluding bathing). A total of 30 accelerometer-derived physical activity measures were categorized into intensity-specific durations, step and cadence summaries, and peak cadence. At FHS, diagnoses of cognitive impairment were established by a review committee based on established criteria. Cox proportional hazard models were used to examine the association with incident cognitive impairment, adjusting for age, gender, education, and accelerometer wear time. Time-dependent area under the receiver operating characteristic curves, derived from random survival forest models, were used to assess predictive performance. Linear regression models were used to evaluate the associations between physical activity measures and 18 neuropsychological test scores.

**Results:**

Among 1212 participants from the FHS Offspring cohort (age: mean 70, SD 8 y; women: 651/1212, 53.71%; follow-up period: mean 9, SD 2 y), 10 physical activity measures, including all peak cadence metrics, were nominally associated with incident cognitive impairment. Higher peak 1-minute cadence (steps/min) was significantly associated with lower risk (hazard ratio 0.82; 95% CI 0.69‐0.97; *P*=.02). Incorporating peak 1-minute cadence into the base model (age, gender, education, and accelerometer wear time) improved the 8-year prediction area under the curve by 10.2%. Peak 1-minute cadence and total steps per day were also associated with better performance on trail making tests A and B.

**Conclusions:**

This study highlights significant associations between accelerometer-based physical activity metrics and both incident cognitive impairment and cognitive test performance. In particular, moderate-intensity movement, as reflected by cadence measures, may serve as a valuable marker for cognitive health and a potential target for early intervention.

## Introduction

### Background

Physical activity is increasingly recognized as a critical determinant of overall health, including cognitive function [1]. It is identified as 1 of 14 modifiable risk factors for cognitive impairment [2]. A growing body of research has shown that regular physical activity is associated with a reduced risk of cognitive decline and enhanced brain function in older adults [3,4]. Meta-analyses further support this association, consistently linking higher levels of physical activity with a lower risk of developing dementia [5-7]. These findings underscore the importance of assessing and monitoring physical activity in older adults to facilitate early detection and intervention efforts targeting cognitive decline.

Despite the value of existing studies, most have relied on self-reported assessments of physical activity [8-11]. While practical and cost-effective for large-scale epidemiological research, self-reported measures are inherently limited by recall bias and subjectivity [12,13]. Recent advancements in digital technology have transformed physical activity assessment by enabling the collection of passive, objective data through wearable devices such as accelerometers. These devices provide high-resolution, continuous data without requiring participant input, thereby reducing burden and minimizing bias associated with memory or self-reporting [14]. Importantly, they capture a broad range of physical activities, including nonexercise behaviors such as household and occupational tasks, offering a more comprehensive picture of daily movement patterns.

One emerging metric from accelerometry is peak cadence, which reflects the highest sustained walking intensity during daily life [15]. Peak cadence has been linked to cardiometabolic risk factors [16], which themselves are known contributors to cognitive decline [17]. Thus, objective physical activity metrics, such as cadence, provide valuable opportunities to more accurately assess physical and cognitive health in real-world settings.

Although an increasing number of studies have begun to explore the link between accelerometer-measured physical activity and cognitive outcomes in older adults [18,19], most have been limited to cross-sectional analyses with modest sample sizes. Understanding how physical activity relates to performance on neuropsychological tests is particularly important, as these tests evaluate cognitive function across multiple domains, such as memory and executive functions [20-22]. In addition, given the well-established associations between slower gait speed and cognitive decline [23,24], cadence, a reflection of gait speed in the free-living environment [25], warrants focused investigation. Exploring these associations could yield valuable insights into the pathways through which physical activity supports brain health and help identify activity patterns most beneficial for cognitive preservation. Such insights may inform more targeted and effective interventions to prevent or delay cognitive decline.

### Objectives

This study aims to test the hypothesis that passive, accelerometer-based physical activity and cadence measures can serve as early indicators of cognitive impairment. Leveraging rich, longitudinal data from the Framingham Heart Study (FHS), we examined associations between these objective physical activity metrics and neuropsychological test performance across multiple cognitive domains. We further evaluated their association with incident cognitive impairment to assess their potential as early biomarkers for identifying individuals at elevated risk and informing strategies for prevention and intervention.

## Methods

### Study Population

**Figure 1.**
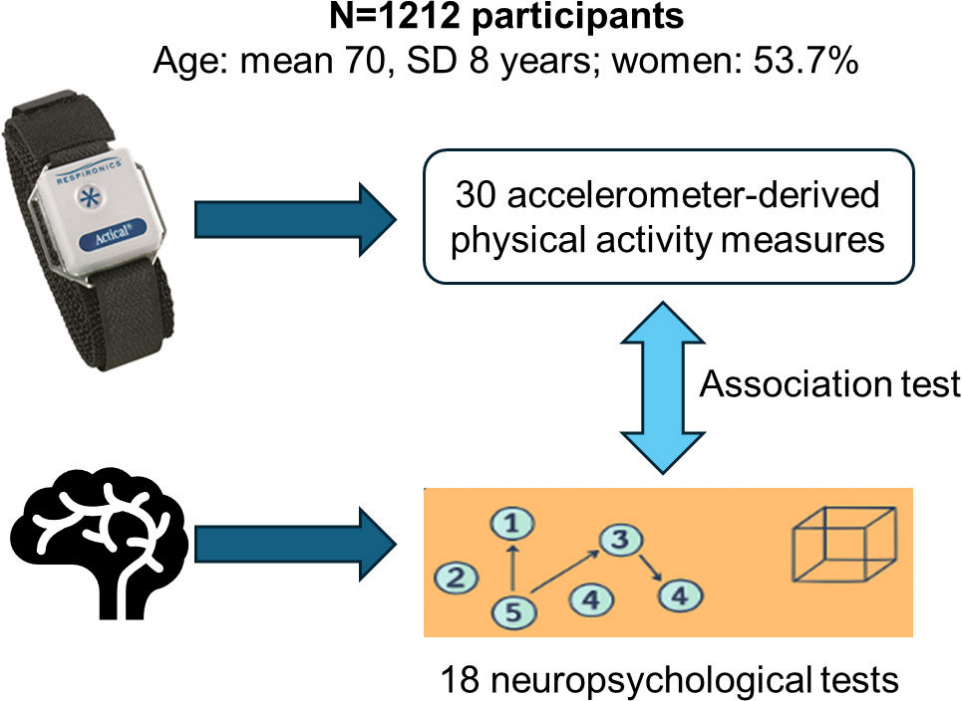
Study design overview.

The FHS is a long-term, community-based cohort study that began in 1948 in Framingham, Massachusetts, and now spans multiple generations. Cognitive assessments have been conducted as part of the FHS since 1976 [26,27]. The Offspring cohort, established in 1972, enrolled 5124 individuals comprising the children of the original cohort members and their spouses [28]. Participants in the Offspring cohort undergo in-person health examinations every 4 to 8 years [29]. During examination cycle 9 (2011‐2014), participants were instructed to wear an Actical accelerometer on their right hip using a waist-worn belt for monitoring physical activity. Participants were asked to wear the device during waking hours, excluding sleep, and were provided with the option to start immediately or at a later time if personal circumstances (eg, illness, surgery, or travel) precluded immediate use. Devices were mailed to participants when needed. After the monitoring period, participants returned the devices to FHS staff, who processed the data, checked for anomalies, and prepared the units for future use. A total of 1646 Offspring participants with reprocessed physical activity data were initially included, with 76 participants excluded for not meeting the criterion of at least 3 adherent days (≥10 h/d). The analysis focused on the 1212 participants with a time interval of ≤5 years between the physical activity measurement date and neuropsychological examination date. Figure 1 presents an overview of this study.

### Ethical Considerations

The study was approved by the institutional review board at Boston University Medical Center (protocol H-32132). All participants provided written informed consent. The original consent or institutional review board approval permitted secondary data analysis without the need for additional consent. All data used in this study were deidentified. Participants did not receive any compensation.

### Accelerometry Data Acquisition

Physical activity was monitored using the Actical omnidirectional accelerometer (model #198-0200-00; Philips Respironics). This device is compact and waterproof (28×27×10 mm and 17 g), designed to detect accelerations from 0.05 to 2.0 g within a frequency range of 0.35 to 3.5 Hz. The device recorded acceleration and deceleration magnitudes in 30-second epochs, categorizing the data into “counts” or “steps.” At the FHS, data were processed using Kinesoft software (version 3.3.63; Kinesoft, Inc) under a standardized protocol to ensure high data quality [30].

### Accelerometer Data Reduction

Nonwear periods were excluded using the Choi algorithm [31]. A day was considered adherent if the device was worn for at least 10 hours, across a minimum of 3 days. To normalize differences in wearing time, we excluded a 6-hour window from each 24-hour period during which the fewest counts were recorded, representing a potential sleeping period [30]. Activity levels were categorized by intensity: sedentary time (≤100 counts/min), moderate activity (1535‐3961 counts/min), and vigorous activity (≥3962 counts/min) [32,33]. To further account for varying wear times, sedentary minutes were normalized to an 18-hour wear period. Average daily step counts were calculated across adherent days, with values >20,000 adjusted to mitigate the influence of outliers. A total of 30 physical activity measures were analyzed in this study (Table S1 in Multimedia Appendix 1).

### Neuropsychological Assessment

Previous studies have detailed the procedures used for administering the neuropsychological tests at FHS [34,35]. This study included the 18 neuropsychological tests (Table S2 in Multimedia Appendix 1), assessing various cognitive functions, such as attention and concentration as well as abstract reasoning [36-41]. Trail making test part B minus part A (trails B-A) was calculated as an additional neuropsychological measure to provide a clearer measure of executive function. This derived score isolates the executive abilities necessary to complete trails B by removing the basic sequencing and psychomotor components that are common to both trails A and trails B. The distributions of the Hooper Visual Organization Test, trails A, trails B, and trails B-A were normalized by applying a natural log transformation. To ensure consistency in interpretation, these transformed values were adjusted so that higher scores indicate better performance on the tasks [42].

### Ascertainment of Cognitive Impairment

In this study, cognitive impairment was categorized as dementia or mild cognitive impairment (MCI). At FHS, diagnoses of cognitive impairment were established by a review committee comprising at least 1 neurologist and 1 neuropsychologist [43]. The diagnosis of dementia followed the criteria established in the *Diagnostic and Statistical Manual of Mental Disorders,* 4th Edition [44]. MCI was defined as cognitive decline in one or more domains without functional impairment or meeting dementia criteria [45]. Detailed information about the diagnostic procedures and cognitive surveillance process at FHS is available in prior research [46]. Prevalent cognitive impairment cases at baseline were excluded. Incident cognitive impairment cases were defined as participants who were cognitively intact at baseline but were subsequently diagnosed with cognitive impairment during the follow-up period. For participants diagnosed with incident cognitive impairment, follow-up time was calculated from the date of physical activity measurement to the earliest documented date for dementia or the impairment date for MCI. For participants who did not develop cognitive impairment, follow-up time was censored at the last known contact date or the date of death, whichever came first.

### Statistical Analyses

To evaluate the differences in baseline characteristics between participants with incident cognitive impairment and those who remained cognitively intact, continuous variables were analyzed using the Kruskal-Wallis *H* test, while categorical variables were assessed with the chi-square test. Standardized values of physical activity measures, with a mean of 0 (SD 1), were used in the subsequent analysis. Linear regression models were used to assess the association between each of the 30 physical activity measures and neuropsychological tests adjusted for age, gender, education, average accelerometer wear time on adherent days, and the time interval between neuropsychological and physical activity examination dates. The Cox proportional hazards model was used to evaluate the associations of incident cognitive impairment with each of the 30 physical activity measures, adjusting for age, gender, education, and accelerometer wear time. In this analysis, participants with prevalent cognitive impairment (n=44) or without follow-up information (n=1) were excluded. To account for multiple comparisons, we applied the Bonferroni correction method [47]. The corrected significance threshold was calculated as *P*=.05/30 approximately .002. As a sensitivity analysis, the associations were further adjusted for additional health-related covariates, including BMI, hypertension treatment, lipid treatment, diabetes, cardiovascular diseases, and stroke. To further investigate the impact of physical activity, Kaplan-Meier survival models were fitted for 2 participant groups: those with peak 1-minute cadence ≥80 steps per minute and those with values <80 steps per minute. The cumulative incidence of cognitive impairment was plotted to visualize the differences between the 2 groups over time.

To evaluate the predictive enhancement of physical activity measures for incident cognitive impairment over multiple follow-up time points (2, 4, 6, and 8 y), additional analyses were performed using a random survival forest model, a robust machine learning approach for survival data [48,49]. The baseline model (model 1) included predictors such as age, gender, educational level, and average accelerometer wear time. Building upon this, model 2 incorporated moderate-to-vigorous physical activity (MVPA) minutes per day. Model 3 included total steps per day on model 1. To explore the impact of peak cadence metrics, model 4 extended model 1 by including peak 1-minute cadence. Finally, model 5 extended model 1 by incorporating these 3 measures of physical activity or cadence. Hyperparameter tuning was conducted for each random survival forest model using a grid search strategy across key parameters, including the minimum size of terminal node (5, 10, 15, and 20), number of trees (100, 150, and 200), and number of randomly selected split points (1, 3, and 5). For each fold of the 5-fold cross-validation, the optimal combination of hyperparameters was selected based on the minimum out-of-bag error rate. The tuned model was then used to generate risk predictions on the held-out test data. Model performance was evaluated using the mean time-dependent area under the receiver operating characteristic curve at 2, 4, 6, and 8 years across the 5 folds [50].

## Results

### Cohort Description

Our study included 1212 participants from the FHS Offspring cohort (age: mean 70, SD 8 y; women: n=651, 53.71%) for analyses examining the association between physical activity and neuropsychological test performance. For analyses related to incident cognitive impairment and prediction modeling, 96.29% (1167/1212) participants were included after excluding individuals with prevalent cognitive impairment at baseline or without follow-up data. During a mean follow-up period of 9 (SD 2) years, 11.23% (131/1167) participants were diagnosed with cognitive impairment. Baseline characteristics significantly varied between those who developed cognitive impairment and those who remained cognitively intact. Specifically, differences were observed in age, education, daily time spent in MVPA, total steps per day, and whether the peak 1-minute cadence reached or exceeded 80 steps per minute (*P*<.05). No significant differences were observed in terms of gender, BMI, diabetes, or prevalent cardiovascular disease. Details of the sample characteristics are presented in Table 1.

**Table 1.** Baseline sample characteristics (N=1212).

Variable	Study sample 1^a^	Study sample 2^b^ (n=1167)		
		Incident cognitive impairment (n=131)	Cognitively intact (n=1036)	*P* value^c^
Age (y), mean (SD)	70 (8)	75 (7)	69 (8)	<.001
Gender, n (%)				.95
Women	651 (53.7)	70 (53.4)	481 (53.6)	
Men	561 (46.3)	61 (46.6)	555 (46.4)	
Education, n (%)				.004
No high school	27 (2.2)	5 (3.8)	16 (1.5)	
High school	278 (22.9)	39 (29.8)	220 (21.2)	
Some college	334 (27.6)	41 (31.3)	280 (27)	
College and higher	573 (47.3)	46 (35.1)	520 (50.2)	
BMI, mean (SD)	28.0 (4.9)	27.7 (4.6)	28.1 (4.9)	.57
Diabetes, n (**%**)	155 (12.8)	23 (17.6)	124 (12)	.10
Cardiovascular disease, n (**%**)	151 (12.5)	16 (12.2)	128 (12.4)	.07
Physical activity, mean (SD)				
Minutes per day spent in sedentary physical activity	654.8 (81.8)	661.7 (82.6)	652.6 (81.5)	.30
Minutes per day spent in moderate-to-vigorous physical activity	13.5 (22.9)	8.2 (13.5)	14.6 (24.1)	<.001
Total steps per day	5993.9 (3731.2)	4839.1 (3282.1)	6256.1 (3764.4)	<.001
Peak 1-min cadence ≥80^c^, n (%)	991 (81.8)	88 (67.2)	879 (84.8)	<.001

a Study sample 1 was used to investigate the associations between physical activity measures and neuropsychological test performance.

b After excluding participants with prevalent cognitive impairments and those without follow-up information, study sample 2 was used for the analysis of associations between physical activity measures and incident cognitive impairments, as well as for predictive modeling analyses.

c A binary variable indicating whether it is ≥80 steps per minute.

### Association Between Physical Activity and Incident Cognitive Impairment

**Figure 2.**
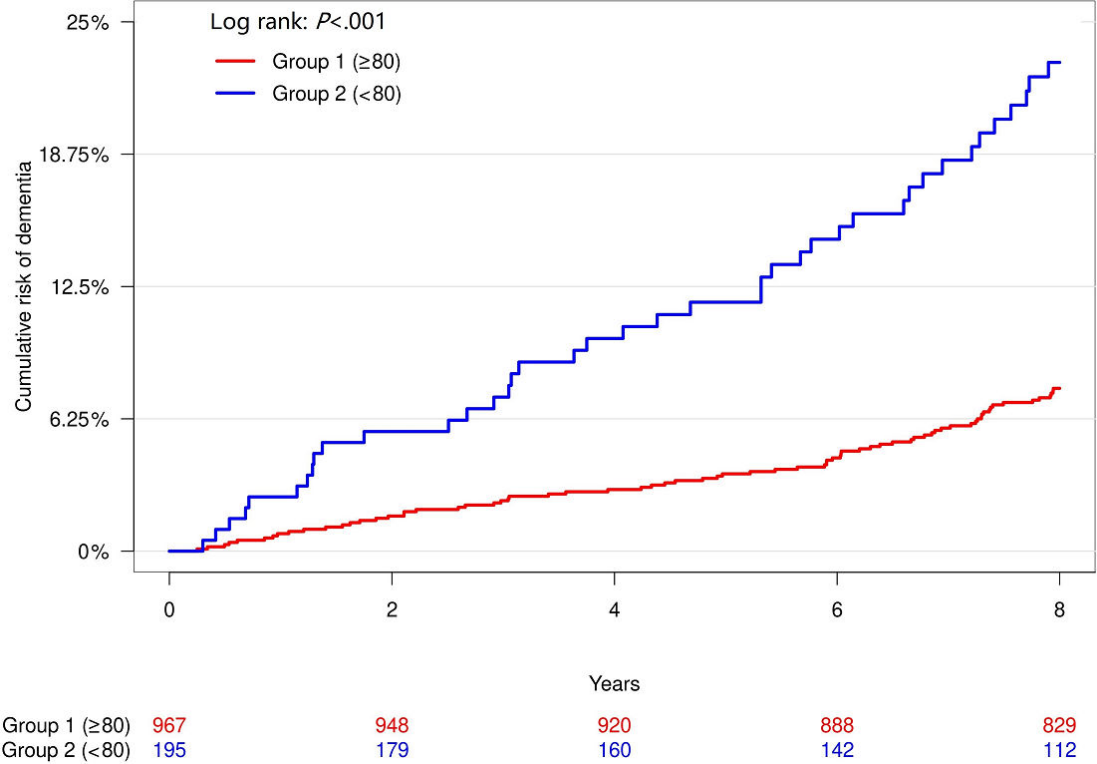
Unadjusted cumulative incidence of cognitive impairment. Group 1 includes participants with the peak 1-minute cadence ≥80 steps per minute, and group 2 includes participants with this measure of <80 steps per minute.

A total of 10 physical activity measures, including all peak cadence metrics, demonstrated nominal significance in their association with incident cognitive impairment, after adjusting for age, gender, educational level, and average accelerometer wear time (Table 2). One SD increase in the total steps per minute taken from the highest 10 minutes (not necessarily consecutive) was significantly associated with a 22% lower risk of incident cognitive impairment (hazard ratio 0.78, 95% CI 0.65‐0.94; *P*=.01). Results based on raw (unstandardized) physical activity measures are presented in Table S3 in Multimedia Appendix 1. In addition, Table S4 in Multimedia Appendix 1 shows the association results after further adjustment for BMI and comorbidities, which remained consistent with the primary analyses. Figure 2 displays the cumulative incidence of cognitive impairment in an unadjusted analysis comparing participants with peak 1-minute cadence ≥80 steps per minute (group 1) versus those with <80 steps per minute (group 2). By year 8, the cumulative incidence was 23.1% in group 2, significantly higher than the 7.7% observed in group 1 (*P*<.001).

**Table 2.** The association between physical activity measures and incident cognitive impairment. Regression models adjusted for age, gender, educational level, and average accelerometer wear time.

Physical activity measure	HR^a^ (95% CI)		*P* value
Intensity-specific durations			
Counts per day	0.66 (0.43-1.02)		.06
Counts per minute	0.67 (0.44-1.00)		.05
Minutes per day spent in sedentary physical activity	1.15 (0.91-1.46)		.24
Minutes per day spent in light physical activity	0.94 (0.76-1.16)		.55
Minutes per day spent in moderate physical activity	0.78 (0.61-1.01)		.06
Minutes per day spent in vigorous physical activity	0.77 (0.46-1.27)		.31
Minutes per day spent in moderate-to-vigorous physical activity	0.72 (0.52-1.00)		.05
Total minutes per day accumulated in sedentary intensity activity	1.16 (0.92-1.46)		.22
Total minutes per day accumulated in moderate-to-vigorous intensity activity	0.78 (0.57-1.07)		.12
Step and cadence summaries			
Steps per day	0.80 (0.65-1.00)		.049
Steps per day with steps per minute <500 removed	0.77 (0.59-0.99)		.04
Steps per minute	0.80 (0.65-0.99)		.04
Steps per minute with steps per minute <500 removed	0.76 (0.59-0.97)		.03
Minutes per day spent in cadence of 0 steps per minute	1.18 (0.96-1.46)		.12
Minutes per day spent in the cadence range of 1-39 steps per minute	0.95 (0.79-1.15)		.62
Minutes per day spent in the cadence range of 40-99 steps per minute	0.81 (0.64-1.03)		.09
Minutes per day spent in cadence of at least 40 steps per minute	0.79 (0.62-1.00)		.045
Minutes per day spent in cadence of at least 100 steps per minute	0.85 (0.67-1.06)		.15
Minutes per day spent in the cadence range of 1-9 steps per minute	1.08 (0.90-1.30)		.41
Minutes per day spent in the cadence range of 1-19 steps per minute	1.01 (0.84-1.21)		.93
Minutes per day spent in the cadence range of 20-39 steps per minute	0.89 (0.73-1.08)		.23
Minutes per day spent in the cadence range of 40-59 steps per minute	0.87 (0.71-1.08)		.21
Minutes per day spent in the cadence range of 60-79 steps per minute	0.78 (0.58-1.03)		.08
Minutes per day spent in the cadence range of 80-99 steps per minute	0.75 (0.55-1.02)		.06
Minutes per day spent in the cadence range of 100-119 steps per minute	0.84 (0.67-1.06)		.14
Peak cadence			
Peak 1-minute cadence (steps/min)	0.82 (0.69-0.97)		.02
Peak 5-minute cadence (steps/min)	0.79 (0.67-0.95)		.01
Peak 10-minute cadence (steps/min)	0.78 (0.65-0.94)		.01
Peak 30-minute cadence (steps/min)	0.79 (0.65-0.96)		.02
Peak 60-minute cadence (steps/min)	0.80 (0.66-0.97)		.02

a HR: hazard ratio.

**Figure 3.**
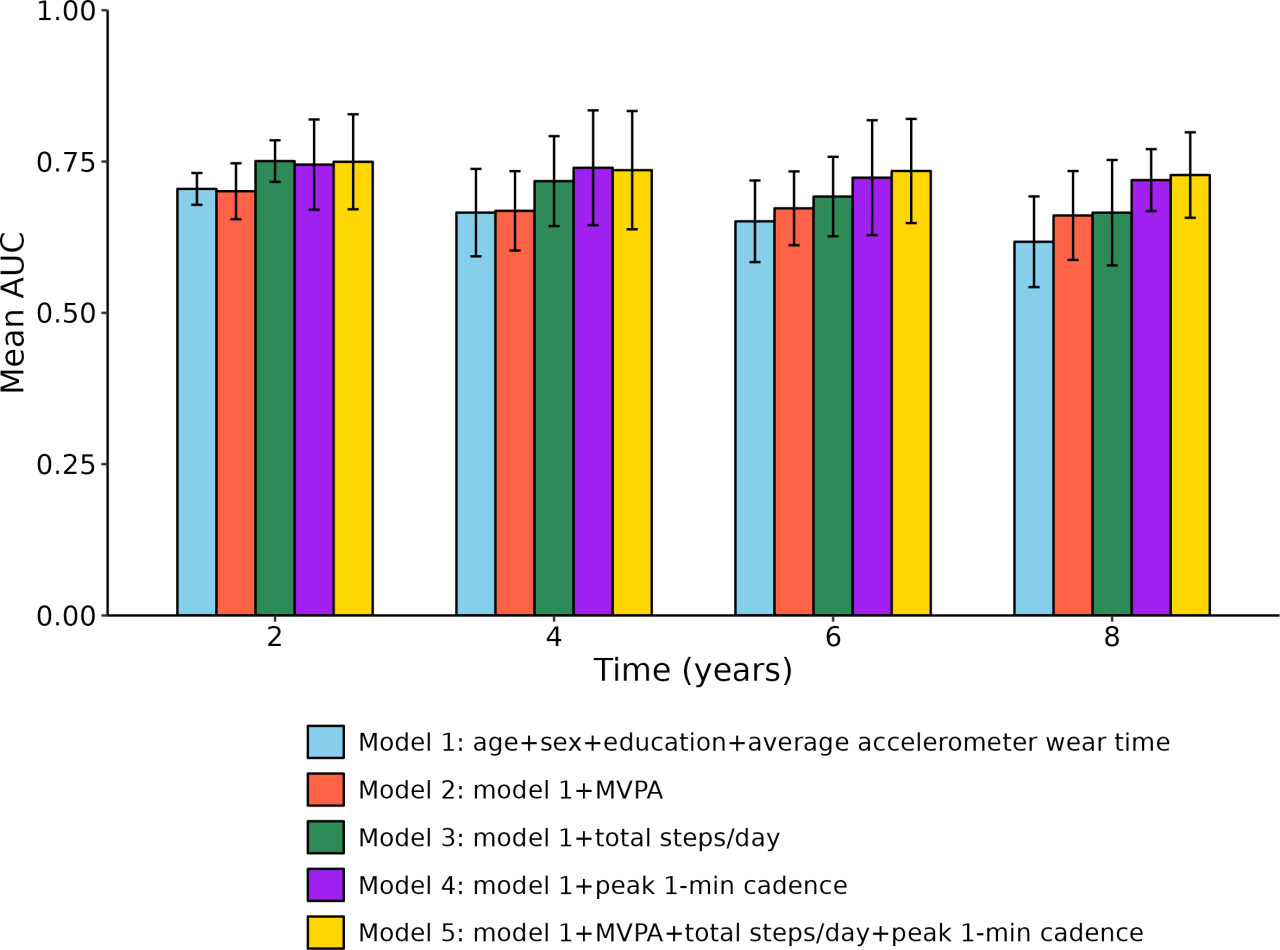
Time-dependent area under the curve (AUC) of 4 models to predict incident cognitive impairment within 2, 4, 6, and 8 years. MVPA: moderate-to-vigorous physical activity.

Figure 3 illustrates the predictive performance of 5 models for incident cognitive impairment across follow-up periods of 2, 4, 6, and 8 years. Model 1, which included only demographic variables and accelerometer wear time, served as the baseline and achieved its best performance at 2 years (area under the curve [AUC]: mean 0.705, SD 0.026) but showed a decline over time, with a mean AUC of 0.617 (SD 0.075) at 8 years. In contrast, models 3 through 5, which incorporated measures of physical activity and cadence, consistently outperformed the baseline model at all time points. Model 4, which extended model 1 by including peak 1-minute cadence, showed improved performance with a mean AUC of 0.740 (SD 0.095) at 4 years. Model 5, which incorporated all 3 physical activity measures—peak 1-minute cadence, MVPA, and steps per day—demonstrated the highest predictive accuracy at longer follow-up intervals, with mean AUCs of 0.734 (SD 0.086) at 6 years and 0.728 (SD 0.071) at 8 years. However, models including only peak 1-minute cadence performed nearly as well, suggesting that this metric alone may offer stronger and more sustained predictive power for cognitive impairment than other physical activity measures.

### Association Between Physical Activity and Neuropsychological Test Performance

Table S2 in Multimedia Appendix 1 presents the associations of physical activity and cadence measures with neuropsychological tests after Bonferroni correction for multiple tests and adjusting for age, gender, educational level, and average accelerometer wear time. Among the 30 measures of physical activity and cadence analyzed, 16 (53%) were significantly associated with neuropsychological tests, each being associated with at least 2 neuropsychological tests after Bonferroni correction. These measures primarily belong to 3 categories: durations spent at specific intensity cut points, step and cadence summaries, and peak cadence. Notably, peak 30-minute cadence was significantly associated with 4 neuropsychological tests (trails B, trails A, trails B-A, and controlled word association test), with the strongest association observed for trails B (β=.07, SE 0.01; *P*<.001). Trails A and B demonstrated significant associations with multiple physical activity measures. Additional analyses adjusting for BMI and comorbidities (Table S5 in Multimedia Appendix 1) yielded results consistent with the primary findings, suggesting robustness of the observed associations.

## Discussion

### Principal Findings

This study examined the association of 30 waist-worn accelerometer–derived physical activity and cadence measures with incident cognitive impairment. A total of 10 measures, including all peak cadence metrics, demonstrated nominal significance. We also evaluated the added predictive value of these measures of physical activity and cadence for predicting incident cognitive impairment. Notably, incorporating peak 1-minute cadence into a model that included age, gender, education, and accelerometer wear time increased the 8-year prediction AUC by 10.2%. Further adjustment for steps per day and MVPA contributed minimal additional predictive value.

Our study underscored the significant role of physical activity in maintaining cognitive health and its potential use as a predictor of cognitive impairment. These findings align with a substantial body of literature suggesting that physical activity is a critical factor influencing cognitive health [1,3,4,51]. Unlike most prior studies that have relied on self-reported physical activity, which is susceptible to biases and inaccuracies, we used accelerometer-derived measures to provide a more objective and precise assessment of daily activity. This approach not only validates and extends previous research but also allows for a more nuanced understanding of how activity intensity and cadence patterns relate to cognitive functions. Among the neuropsychological tests, trails A (connecting numbered circles in sequence) and trails B (alternating between numbers and letters, eg, 1, A, 2, and B) [52] were most strongly associated with multiple physical activity measures. Trails B, in particular, showed the strongest and most consistent associations across all step, cadence summary, and peak cadence metrics. These results are consistent with prior studies highlighting the sensitivity of trail making tests to variations in physical activity [53-55].

In our longitudinal analysis, 10 physical activity measures, including all measures of peak cadence, were significantly associated with reduced risk of incident cognitive impairment. Cadence, a spatiotemporal parameter of gait, reflects walking speed and the intensity of physical activity in free-living environments [25]. Slower gait speed has been linked to cognitive decline and increased dementia risk [23,24]. When combined with memory impairments, reduced gait speed exhibits an even stronger association with dementia risk, emphasizing its potential as an early marker of cognitive decline [56]. Evidence also suggests that cognitive performance improvements can be achieved following a single 30-minute walking session at 100 steps per minute in older adults [57].

Several biological mechanisms have been suggested to explain the neuroprotective effects of physical activity, including reduction in β-amyloid accumulation, enhancement of cerebral blood flow and vascular integrity, and modulation of oxidative stress and neuroinflammation [58,59]. Indirect benefits, such as improved sleep, mood, and cardiovascular health, may further support cognitive functions [58,59]. Our results highlight cadence as both a marker of cognitive health and a potential target for interventions aimed at mitigating cognitive decline. Models that included physical activity measures consistently outperformed baseline models across all time points, underscoring their predictive value. However, the specific association between cadence and cognition remains underexplored and warrants further investigation.

### Strengths and Limitations

Our study has several notable strengths. It leveraged a large sample from the FHS Offspring cohort with objective accelerometer-based physical activity assessment, spanning a wide range of metrics, such as activity duration at different intensity levels, step and cadence summaries, and peak cadence. The availability of comprehensive neuropsychological testing enabled a detailed assessment of cognitive function across multiple domains. Furthermore, cognitive outcomes were adjudicated clinically, and participants were followed longitudinally, strengthening our ability to assess the impact of physical activity on future cognitive risk.

Nonetheless, we acknowledged several limitations. First, the majority of FHS Offspring participants are of European ancestry, which may limit the generalizability of our findings to more diverse populations. Future studies should include racially and ethnically diverse cohorts to enhance external validity. In addition, we did not account for social determinants of health, such as socioeconomic status or social support, which may influence physical activity levels. Our inclusion of participants with at least 3 days of accelerometer wear reflects a compromise to balance measurement precision with selection bias. Prior analyses have shown that individuals who did not complete accelerometry exhibited poorer cognitive performance [60]; thus, we opted not to restrict the sample further. However, this may reduce the accuracy of estimates for habitual activity patterns [61]. Finally, the potential for reverse causality remains; lower physical activity may contribute to cognitive decline, but early cognitive changes could also reduce engagement in activity. Longitudinal studies with repeated assessments of both cognition and physical activity are needed to better understand these temporal dynamics.

### Conclusions

In summary, our findings emphasize the value of accelerometer-based physical activity and cadence measures in understanding cognitive health. Moderate movement and walking speed, in particular, appear to play a crucial role in preserving cognitive function and reducing the risk of cognitive impairment. These findings support incorporating objective physical activity monitoring into routine assessments of older adults to facilitate early identification and intervention. Future research should continue to explore the longitudinal trajectories of physical activity and cadence in relation to cognitive decline and refine predictive models to enhance their clinical applicability.

## Supplementary material

10.2196/72946 Multimedia Appendix 1 Tables S1 to S5 and descriptions of physical activity measures used in this study.

## Abbreviations

AUC: area under the curve
FHS: Framingham Heart Study
MCI: mild cognitive impairment
MVPA: moderate-to-vigorous physical activity
