# Supplementary material for: Passive Measures of Physical Activity and Cadence as Early Indicators of Cognitive Impairment: Observational Study
Source: J Med Internet Res. 2025 Sep 12;27:e72946. doi: 10.2196/72946 (PMC12431165; doi:10.2196/72946)
Supplement: Multimedia Appendix 1 [file jmir-v27-e72946-s001.docx]

**Supplemental Table 1.** Description and distribution of physical activity measures used in this study.

| **Physical activity** | **Description** | **Median** | **Q1** | **Q3** |
| --- | --- | --- | --- | --- |
| Intensity-specific durations | Counts per day, counts/day | 86397.38 | 50107.25 | 134506.21 |
|  | Counts per minute, counts/min | 103.03 | 60.75 | 159.66 |
|  | Minutes per day spent in sedentary physical activity, min/day | 654.94 | 603.42 | 711.46 |
|  | Minutes per day spent in light physical activity, min/day | 153.83 | 111.19 | 198.31 |
|  | Minutes per day spent in moderate physical activity, min/day | 6.06 | 1.20 | 15.48 |
|  | Minutes per day spent in vigorous physical activity, min/day | 0.00 | 0.00 | 0.29 |
|  | Minutes per day spent in moderate or vigorous physical activity, min/day | 6.50 | 1.20 | 18.30 |
|  | Total minutes per day accumulated in sedentary intensity activity, min/day | 671.94 | 607.23 | 735.78 |
|  | Total minutes per day accumulated in moderate or vigorous intensity activity, min/day | 0.00 | 0.00 | 9.16 |
| Step and cadence summaries | Steps per day, steps/day | 5368.75 | 3200.07 | 8139.44 |
|  | Steps per day with steps per minute below 500 removed, steps/day | 2632.56 | 1197.93 | 4566.32 |
|  | Steps per minute, steps/min | 6.49 | 3.97 | 9.65 |
|  | Steps per minute with steps per minute below 500 removed, steps/min | 3.18 | 1.51 | 5.43 |
|  | Minutes per day spent in cadence of 0 steps per minute, min/day | 609.31 | 541.85 | 669.62 |
|  | Minutes per day spent in the cadence range of 1-39 steps per minute, min/day | 168.21 | 125.13 | 217.32 |
|  | Minutes per day spent in the cadence range of 40-99 steps per minute, min/day | 32.13 | 16.12 | 53.25 |
|  | Minutes per day spent in cadence of at least 40 steps per minute, min/day | 39.63 | 19.00 | 66.03 |
|  | Minutes per day spent in cadence of at least 100 steps per minute, min/day | 2.50 | 0.43 | 9.87 |
|  | Minutes per day spent in the cadence range of 1-9 steps per minute, min/day | 59.14 | 47.00 | 72.13 |
|  | Minutes per day spent in the cadence range of 1-19 steps per minute, min/day | 115.06 | 91.00 | 143.54 |
|  | Minutes per day spent in the cadence range of 20-39 steps per minute, min/day | 51.75 | 31.29 | 75.19 |
|  | Minutes per day spent in the cadence range of 40-59 steps per minute, min/day | 18.76 | 9.48 | 32.00 |
|  | Minutes per day spent in the cadence range of 60-79 steps per minute, min/day | 7.83 | 3.63 | 13.50 |
|  | Minutes per day spent in the cadence range of 80-99 steps per minute, min/day | 4.00 | 1.71 | 7.52 |
|  | Minutes per day spent in the cadence range of 100-119 steps per minute, min/day | 2.22 | 0.40 | 6.92 |
| Peak cadence | Peak 1 minute cadence*, steps/min | 99.00 | 85.41 | 109.86 |
|  | Peak 5 minute cadence, steps/min | 88.91 | 73.00 | 102.27 |
|  | Peak 10 minute cadence, steps/min | 81.81 | 64.38 | 96.57 |
|  | Peak 30 minute cadence, steps/min | 64.84 | 48.26 | 81.59 |
|  | Peak 60 minute cadence, steps/min | 51.23 | 36.14 | 66.76 |

*991 participants (81.8%) recorded a value of 80 steps/min or higher.

**Supplemental Table 2.** The association between physical activity measures and NP tests.

| **Physical activity** **measure** | **The most significant NP test** | **Beta** | **SE** | ***P* value** | ***N**** |
| --- | --- | --- | --- | --- | --- |
| **Intensity-specific durations** |  |  |  |  |  |
| Minutes per day spent in sedentary physical activity, min/day | Trails A | -0.06 | 0.01 | <.001 | 2 |
| Minutes per day spent in light physical activity, min/day | Trails A | 0.05 | 0.01 | <.001 | 2 |
| Total minutes per day accumulated in sedentary intensity activity, min/day | Trails A | -0.05 | 0.01 | <.001 | 2 |
| **Step and cadence summaries** |  |  |  |  |  |
| Steps per day, steps/day | Trails B | 0.05 | 0.01 | <.001 | 2 |
| Steps per day with steps per minute below 500 removed, steps/day |  | 0.05 | 0.01 | <.001 | 2 |
| Steps per minute, steps/min | Trails B | 0.06 | 0.01 | <.001 | 3 |
| Steps per minute below 500 removed, steps/min | Trails B | 0.06 | 0.01 | <.001 | 2 |
| Minutes per day spent in the cadence range of 40-99 steps per minute, min/day | Trails A | 0.04 | 0.01 | <.001 | 2 |
| Minutes per day spent in cadence of at least 40 steps per minute, min/day | Trails B | 0.05 | 0.01 | <.001 | 2 |
| Minutes per day spent in the cadence range of 20-39 steps per minute, min/day | Trails A | 0.04 | 0.01 | <.001 | 2 |
| Minutes per day spent in the cadence range of 40-59 steps per minute, min/day | Trails A | 0.06 | 0.01 | <.001 | 2 |
| **Peak cadence** |  |  |  |  |  |
| Peak 1 minute cadence, steps/min | Trails B | 0.08 | 0.01 | <.001 | 3 |
| Peak 5 minute cadence, steps/min | Trails B | 0.08 | 0.01 | <.001 | 3 |
| Peak 10 minute cadence, steps/min | Trails B | 0.08 | 0.01 | <.001 | 3 |
| Peak 30 minute cadence, steps/min | Trails B | 0.07 | 0.01 | <.001 | 4 |
| Peak 60 minute cadence, steps/min | Trails B | 0.07 | 0.01 | <.001 | 4 |

Note: The NP tests used in this study included (1) Wechsler Memory Scale (WMS) - Logical Memory Immediate Recall, (2) Delayed Recall, (3) Recognition; (4) WMS Paired Associate Learning Immediate Recall, (5) Delayed Recall, (6) Recognition; (7) WMS Visual Reproduction Immediate Recall, (8) Delayed Recall, (9) Recognition; (10) WMS Digit Span Forward and (11) Backward; (12) Wechsler Adult Intelligence Scale (WAIS) Similarities test; (13) Boston Naming Test 30-item version; (14) Controlled Word Association Test, (15) Category Naming; (16) Trail Making Test Part A and (17) Part B (Trails A and B); and (18) the Hooper Visual Organization Test. The association analysis was adjusted for age, sex, education, accelerometer wear time, and the time interval between NP and physical activity examination dates. SE: standard error.

***N represents the number of NP tests significantly associated with the corresponding physical activity measure after Bonferroni correction for multiple testing.

**Supplemental Table 3**. The association between physical activity measures and incident cognitive impairment. The raw values of physical activity measures were used.

| **Physical activity measure** | **HR** | **95% CI** | | ***P* value** |
| --- | --- | --- | --- | --- |
| **Intensity-specific durations** |  |  |  |  |
| Counts per day, counts/day | 1.00 | 1.00 | 1.00 | 0.06 |
| Counts per minute, counts/min | 1.00 | 0.99 | 1.00 | 0.05 |
| Minutes per day spent in sedentary physical activity, min/day | 1.00 | 1.00 | 1.00 | 0.24 |
| Minutes per day spent in light physical activity, min/day | 1.00 | 1.00 | 1.00 | 0.55 |
| Minutes per day spent in moderate physical activity, min/day | 0.98 | 0.97 | 1.00 | 0.06 |
| Minutes per day spent in vigorous physical activity, min/day | 0.98 | 0.93 | 1.02 | 0.31 |
| Minutes per day spent in moderate or vigorous physical activity, min/day | 0.99 | 0.97 | 1.00 | 0.05 |
| Total minutes per day accumulated in sedentary intensity activity, min/day | 1.00 | 1.00 | 1.00 | 0.22 |
| Total minutes per day accumulated in moderate or vigorous intensity activity, min/day | 0.99 | 0.97 | 1.00 | 0.12 |
| **Step and cadence summaries** |  |  |  |  |
| Steps per day, steps/day (per 500-step/day increase) | 0.97 | 0.94 | 1.00 | **0.049** |
| Steps per day with steps per minute below 500 removed, steps/day (per 500-step/day increase) | 0.95 | 0.91 | 1.00 | **0.04** |
| Steps per minute, steps/min | 0.95 | 0.91 | 1.00 | **0.04** |
| Steps per minute with steps per minute below 500 removed, steps/min | 0.92 | 0.86 | 0.99 | **0.03** |
| Minutes per day spent in cadence of 0 steps per minute, min/day | 1.00 | 1.00 | 1.00 | 0.12 |
| Minutes per day spent in the cadence range of 1-39 steps per minute, min/day | 1.00 | 1.00 | 1.00 | 0.62 |
| Minutes per day spent in the cadence range of 40-99 steps per minute, min/day | 0.99 | 0.99 | 1.00 | 0.09 |
| Minutes per day spent in cadence of at least 40 steps per minute, min/day (per 5-min/day increase) | 0.97 | 0.94 | 1.00 | **0.045** |
| Minutes per day spent in cadence of at least 100 steps per minute, min/day | 0.99 | 0.97 | 1.00 | 0.15 |
| Minutes per day spent in the cadence range of 1-9 steps per minute, min/day | 1.00 | 0.99 | 1.01 | 0.41 |
| Minutes per day spent in the cadence range of 1-19 steps per minute, min/day | 1.00 | 1.00 | 1.00 | 0.93 |
| Minutes per day spent in the cadence range of 20-39 steps per minute, min/day | 1.00 | 0.99 | 1.00 | 0.23 |
| Minutes per day spent in the cadence range of 40-59 steps per minute, min/day | 0.99 | 0.98 | 1.00 | 0.21 |
| Minutes per day spent in the cadence range of 60-79 steps per minute, min/day | 0.98 | 0.95 | 1.00 | 0.08 |
| Minutes per day spent in the cadence range of 80-99 steps per minute, min/day | 0.96 | 0.92 | 1.00 | 0.06 |
| Minutes per day spent in the cadence range of 100-119 steps per minute, min/day | 0.98 | 0.96 | 1.01 | 0.14 |
| **Peak cadence** |  |  |  |  |
| Peak 1 minute cadence, steps/min (per 5-step/min increase) | 0.95 | 0.91 | 0.99 | **0.02** |
| Peak 5 minute cadence, steps/min (per 5-step/min increase) | 0.95 | 0.91 | 0.99 | **0.01** |
| Peak 10 minute cadence, steps/min (per 5-step/min increase) | 0.95 | 0.91 | 0.99 | **0.01** |
| Peak 30 minute cadence, steps/min (per 5-step/min increase) | 0.95 | 0.92 | 0.99 | **0.02** |
| Peak 60 minute cadence, steps/min (per 5-step/min increase) | 0.95 | 0.91 | 0.99 | **0.02** |

**Supplemental Table 4**. The association between physical activity measures and incident cognitive impairment with additional adjustment for BMI and comorbidities.

| **Physical activity measure** | **HR** | **95% CI** | | ***P* value** |
| --- | --- | --- | --- | --- |
| **Intensity-specific durations** |  |  |  |  |
| Counts per day, counts/day | 0.62 | 0.39 | 0.99 | **0.04** |
| Counts per minute, counts/min | 0.63 | 0.41 | 0.97 | **0.03** |
| Minutes per day spent in sedentary physical activity, min/day | 1.17 | 0.92 | 1.51 | 0.20 |
| Minutes per day spent in light physical activity, min/day | 0.93 | 0.75 | 1.15 | 0.50 |
| Minutes per day spent in moderate physical activity, min/day | 0.77 | 0.59 | 1.00 | 0.05 |
| Minutes per day spent in vigorous physical activity, min/day | 0.75 | 0.45 | 1.25 | 0.27 |
| Minutes per day spent in moderate or vigorous physical activity, min/day | 0.70 | 0.50 | 0.99 | **0.04** |
| Total minutes per day accumulated in sedentary intensity activity, min/day | 1.17 | 0.92 | 1.49 | 0.20 |
| Total minutes per day accumulated in moderate or vigorous intensity activity, min/day | 0.76 | 0.55 | 1.05 | 0.10 |
| **Step and cadence summaries** |  |  |  |  |
| Steps per day, steps/day | 0.78 | 0.62 | 0.98 | **0.04** |
| Steps per day with steps per minute below 500 removed, steps/day | 0.74 | 0.57 | 0.97 | **0.03** |
| Steps per minute, steps/min | 0.78 | 0.63 | 0.97 | **0.03** |
| Steps per minute with steps per minute below 500 removed, steps/min | 0.74 | 0.57 | 0.96 | **0.02** |
| Minutes per day spent in cadence of 0 steps per minute, min/day | 1.21 | 0.98 | 1.50 | 0.08 |
| Minutes per day spent in the cadence range of 1-39 steps per minute, min/day | 0.94 | 0.78 | 1.14 | 0.53 |
| Minutes per day spent in the cadence range of 40-99 steps per minute, min/day | 0.80 | 0.63 | 1.03 | 0.08 |
| Minutes per day spent in cadence of at least 40 steps per minute, min/day | 0.77 | 0.60 | 0.98 | **0.04** |
| Minutes per day spent in cadence of at least 100 steps per minute, min/day | 0.84 | 0.66 | 1.05 | 0.13 |
| Minutes per day spent in the cadence range of 1-9 steps per minute, min/day | 1.08 | 0.89 | 1.30 | 0.44 |
| Minutes per day spent in the cadence range of 1-19 steps per minute, min/day | 1.00 | 0.83 | 1.20 | 0.99 |
| Minutes per day spent in the cadence range of 20-39 steps per minute, min/day | 0.87 | 0.71 | 1.06 | 0.17 |
| Minutes per day spent in the cadence range of 40-59 steps per minute, min/day | 0.86 | 0.69 | 1.07 | 0.19 |
| Minutes per day spent in the cadence range of 60-79 steps per minute, min/day | 0.76 | 0.57 | 1.03 | 0.08 |
| Minutes per day spent in the cadence range of 80-99 steps per minute, min/day | 0.74 | 0.54 | 1.02 | 0.06 |
| Minutes per day spent in the cadence range of 100-119 steps per minute, min/day | 0.84 | 0.67 | 1.06 | 0.14 |
| **Peak cadence** |  |  |  |  |
| Peak 1 minute cadence, steps/min | 0.80 | 0.67 | 0.95 | **0.01** |
| Peak 5 minute cadence, steps/min | 0.77 | 0.64 | 0.92 | **0.005** |
| Peak 10 minute cadence, steps/min | 0.76 | 0.63 | 0.91 | **0.004** |
| Peak 30 minute cadence, steps/min | 0.77 | 0.63 | 0.93 | **0.008** |
| Peak 60 minute cadence, steps/min | 0.77 | 0.63 | 0.95 | **0.012** |

Regression models adjusted for age, sex, educational level, average accelerometer wear time, BMI, hypertension treatment, lipid treatment, diabetes, cardiovascular diseases, and stroke.

**Supplemental Table 5.** The association between physical activity measures and NP tests with additional adjustment for BMI and comorbidities.

| **Physical activity** **measure** | **The most significant NP test** | **Beta** | **SE** | ***P* value** | ***N**** |
| --- | --- | --- | --- | --- | --- |
| **Intensity-specific durations** |  |  |  |  |  |
| Minutes per day spent in sedentary physical activity, min/day | Trails A | -0.06 | 0.01 | <.001 | 2 |
| Minutes per day spent in light physical activity, min/day | Trails A | 0.05 | 0.01 | <.001 | 2 |
| Total minutes per day accumulated in sedentary intensity activity, min/day | Trails A | -0.05 | 0.01 | <.001 | 2 |
| **Step and cadence summaries** |  |  |  |  |  |
| Steps per day, steps/day | Trails B | 0.05 | 0.01 | <.001 | 2 |
| Steps per day with steps per minute below 500 removed, steps/day |  |  |  |  |  |
| Steps per minute, steps/min | Trails B | 0.06 | 0.01 | <.001 | 2 |
| Steps per minute below 500 removed, steps/min | Trails B | 0.05 | 0.01 | <.001 | 1 |
| Minutes per day spent in the cadence range of 40-99 steps per minute, min/day | Trails A | 0.04 | 0.01 | <.001 | 1 |
| Minutes per day spent in cadence of at least 40 steps per minute, min/day | Trails B | 0.05 | 0.01 | <.001 | 1 |
| Minutes per day spent in the cadence range of 20-39 steps per minute, min/day | Trails A | 0.04 | 0.01 | <.001 | 2 |
| Minutes per day spent in the cadence range of 40-59 steps per minute, min/day | Trails A | 0.04 | 0.01 | <.001 | 2 |
| **Peak cadence** |  |  |  |  |  |
| Peak 1 minute cadence, steps/min | Trails B | 0.08 | 0.02 | <.001 | 3 |
| Peak 5 minute cadence, steps/min | Trails B | 0.08 | 0.02 | <.001 | 3 |
| Peak 10 minute cadence, steps/min | Trails B | 0.08 | 0.02 | <.001 | 3 |
| Peak 30 minute cadence, steps/min | Trails B | 0.07 | 0.02 | <.001 | 3 |
| Peak 60 minute cadence, steps/min | Trails B | 0.07 | 0.02 | <.001 | 3 |

Note: The association analysis was adjusted for age, sex, education, accelerometer wear time, the time interval between NP and physical activity examination dates, BMI, hypertension treatment, lipid treatment, diabetes, cardiovascular diseases, and stroke. SE: standard error.

***N represents the number of NP tests significantly associated with the corresponding physical activity measure after Bonferroni correction for multiple testing.
